# Automated Precancerous Lesion Screening Using an Instance Segmentation Technique for Improving Accuracy

**DOI:** 10.3390/s22155489

**Published:** 2022-07-22

**Authors:** Patiyus Agustiansyah, Siti Nurmaini, Laila Nuranna, Irfannuddin Irfannuddin, Rizal Sanif, Legiran Legiran, Muhammad Naufal Rachmatullah, Gavira Olipa Florina, Ade Iriani Sapitri, Annisa Darmawahyuni

**Affiliations:** 1 Doctoral Program, Biology Science, Faculty of Medicine, Universitas Sriwijaya, Palembang 30139, Indonesia; fatiyusagustiansyah@gmail.com; 2 Division of Oncology-Gynecology, Department of Obstetrics and Gynecology, Mohammad Hoesin General Hospital, Palembang 30126, Indonesia; 3 Intelligent System Research Group, Faculty of Computer Science, Universitas Sriwijaya, Palembang 30139, Indonesia; naufalrachmatullah@gmail.com (M.N.R.); gaviraflorina.01@gmail.com (G.O.F.); adeirianisapitri13@gmail.com (A.I.S.); riset.annisadarmawahyuni@gmail.com (A.D.); 4 Obstetrics & Gynecology Department, Faculty of Medicine, University of Indonesia, Jakarta 10430, Indonesia; laila.nuranna@ui.ac.id; 5 Obstetrics & Gynecology Department, Faculty of Medicine, Universitas Sriwijaya, Palembang 30139, Indonesia; irfan.md@unsri.ac.id (I.I.); rizalsanif@fk.unsri.ac.id (R.S.); dr.legiran@fk.unsri.ac.id (L.L.)

**Keywords:** instance segmentation, squamocolumnar junction, columnar area, acetowhite lesions, visual inspection of acetic acid

## Abstract

Precancerous screening using visual inspection with acetic acid (VIA) is suggested by the World Health Organization (WHO) for low–middle-income countries (LMICs). However, because of the limited number of gynecological oncologist clinicians in LMICs, VIA screening is primarily performed by general clinicians, nurses, or midwives (called medical workers). However, not being able to recognize the significant pathophysiology of human papilloma virus (HPV) infection in terms of the columnar epithelial-cell, squamous epithelial-cell, and white-spot regions with abnormal blood vessels may be further aggravated by VIA screening, which achieves a wide range of sensitivity (49–98%) and specificity (75–91%); this might lead to a false result and high interobserver variances. Hence, the automated detection of the columnar area (CA), subepithelial region of the squamocolumnar junction (SCJ), and acetowhite (AW) lesions is needed to support an accurate diagnosis. This study proposes a mask-RCNN architecture to simultaneously segment, classify, and detect CA and AW lesions. We conducted several experiments using 262 images of VIA+ cervicograms, and 222 images of VIA−cervicograms. The proposed model provided a satisfactory intersection over union performance for the CA of about 63.60%, and AW lesions of about 73.98%. The dice similarity coefficient performance was about 75.67% for the CA and about 80.49% for the AW lesion. It also performed well in cervical-cancer precursor-lesion detection, with a mean average precision of about 86.90% for the CA and of about 100% for the AW lesion, while also achieving 100% sensitivity and 92% specificity. Our proposed model with the instance segmentation approach can segment, detect, and classify cervical-cancer precursor lesions with satisfying performance only from a VIA cervicogram.

## 1. Introduction

Cervical cancer has become a public health concern and global health burden, with approximately 90% of fatalities coming from low–middle-income countries (LMICs) [1]. Following a 27% increase in fatality rate by 2030, the frequency of cervical cancer is expected to increase to 21% [1,2]. Increasing the accurate and thorough coverage screening of cervical cancer precursor lesions in LMICs is a main method for reducing the incidence and fatality rate of the disease [3,4,5,6]. Cervical cancer is a preventable disease caused by an oncogenic form of the human papilloma virus (HPV), which is an oncogenic variant/high-risk type [7]. According to Herf’s pathophysiology of HPV infection [4,5], viral infections originate from the subepithelial region of the squamocolumnar junction (SCJ) and spread into the cervical transformation zone (TJ). To diagnose this condition, clinicians must be able to recognize the columnar area (CA), SCJ subepithelial regions, and acetowhite (AW) lesions. If the subepithelial SCJ region and AW lesion do not overlap, they are defined as condyloma lesions (which are created by low-risk HPV virus) or Nabothian cysts (blocked Nabothian gland). The pathophysiology of HPV infection is a crucial anatomical characteristic for diagnosing cervical-cancer precursor lesions [8,9,10].

Cervical-cancer precursor lesions can progress to invasive carcinoma after about 10–15 years [11,12,13,14]; hence, there is a “golden time” to perform early screening to break the disease’s chain. Currently, precancerous lesion screening consists of various methods, including a Papanicolaou (Pap) smear based on liquid-base cytology (LBC), visual inspection with acetic acid (VIA), visual inspection with Lugol’s iodine (VILI), and HPV DNA test based on genotyping or hybrid capture [14]. Pap smear screening programs have been successful in reducing the burden of cervical cancer in the developed world [3,8]. In LMICs, it is not a feasible option because of the lack of trained cytopathologists, difficulties in following up with screen-positive women, the absence of quality assurance measures, and poor healthcare infrastructures [1,2,8]. VIA screening is recommended by the WHO for LMICs because of its low cost and simple process, and it can be performed in a real-time screening test. In every VIA+ case, cold ablation (cryotherapy) intervention is conducted at the same visit to prevent the loss of patient follow-up [6]. However, VIA has the limitations of low specificity, requiring extensive training, and retraining healthcare providers, especially with the test being subjective and challenging regarding quality control [15,16,17,18,19,20]. Hence, automated VIA screening that can achieve accurate and satisfactory performance is needed.

A diagnostic medical procedure to take pictures of the cervix for interpretation on the basis of VIA produces a cervical digital image named a cervicogram. The cervicogram provides the permanent and objective documentation of normal and abnormal anatomical cervical patterns [4,10]. The automated analysis of these important patterns is a challenging task because of the following [5,7,16]: (i) the presence of artifacts such as shadows in the cervical area during the cervicogram acquisition process, which can be caused by light reflections and can produce a concave shape of the cervix; (ii) the complex variability in cervicogram image data in terms of intensity, shape, the presence of ovules of Nabothi, and areas of immature metaplasia; (iii) variability in the cervicogram content because not all cervical tissue appears in the cervicogram; and (iv) the presence of a narrow dynamic color threshold and the unclear boundaries of the tissue region.

Computer-aided diagnosis (CAD) studies based on an artificial intelligence (AI) approach have produced remarkable results in the medical field [17,18,19,20,21,22,23,24,25]. These technologies can automatically predict abnormalities to support patients’ diagnoses and offer medical personnel a second opinion. CAD-based AI methods include machine learning (ML) and deep learning (DL) [21,22,23,24,25]. They can generate a learning model to enhance diagnostic accuracy in a wide variety of clinical sectors. In precancerous detection, various approaches for analyzing cervicogram images have been investigated, including the use of handicraft characteristics and conventional classification algorithms such as support vector machines (SVMs) [17] and K-nearest neighbor (K-NN) [18]. However, ML cannot automatically conduct feature extraction and still requires human intervention. This limits the generalization model in routine clinical applications because of the significant variability in the pathophysiology of the cervicogram images.

The DL model has been proposed to identify precursor cervical-cancer lesions by segmenting, detecting, or classifying them using feature learning. Compared with ML, deep-learning algorithms possess more powerful learning capabilities and can automatically extract features without extensive data preprocessing or handcrafted feature extraction, rendering them a suitable tool for analyzing the complex structures of high-dimensional data [24]. Several studies have been developed to aid diagnoses here by using cervicography and colposcopy images, which are explored in VIA screening to detect AW lesions as symptoms of precursor cervical cancer. These studies used models employing faster region convolutional neural network (CNN) architectures [21], DL models [22], and CNN architectures [23,24]. All these studies show satisfying results, and our model outperformed other ML models.

However, the processes of segmentation, detection, and classification by using cervicography are separately carried out. It is difficult to analyze in great detail where the position of an AW lesion is because of the classification running in the black-box condition. In addition, the classification of AW lesions requires huge datasets; unfortunately, it is hard to collect VIA+ cervicograms. The success of model evaluation depends on the accurate segmentation of CA and AW lesions with the SCJ. Despite recent improvements in this field, the presence of AW lesions, such as condyloma and nabothian cysts, may cause system misinterpretation and impair accuracy. To improve prediction accuracy, our research focuses on segmenting two key aspects that explain the crossing of CA and AW lesions. The proposed model also detects CA and AW lesions simultaneously. Here, mask region networks were employed using the CNN technique for approaching deep learning to interpret cervicogram images; this also helps in the human side, adding faster learning without vast datasets. The contributions and originality of our study are as follows:

- An accurate segmentation of the CA and AW lesion is proposed based on the pathogenesis of cervical cancer by HPV with high confidence and a high intersection over union (IoU) baseline.
- An instance segmentation model was developed with a mask-RCNN architecture to simultaneously segment, classify, and detect AW lesions in VIA+ and VIA−.
- The proposed model was validated within a real clinical setting to ensure that the model could be trusted in terms of false-positive and false-negative predictive values.

The current paper is structured as follows: In Section 2, we present the research methods proposed for cervical-cancer precursor-lesion detection. In Section 3, we present the results of applying the methods and a discussion of these results. Lastly, in Section 4, we draw conclusions.

## 2. Materials and Methods

**Figure 1:**
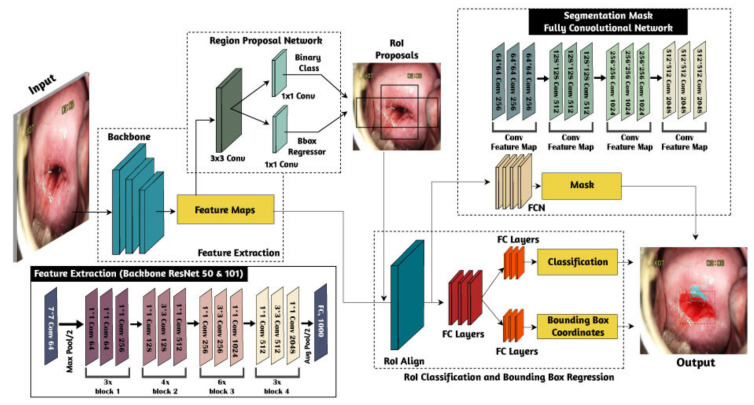
The proposed methodology of instance segmentation for automate screening of VIA cervicograms based on cervical anatomy with CA and AW lesions.

The entire methodology was divided into five main processes: data acquisition, data preparation, annotation labels, DL model training, and model evaluation. The DL model was mainly used to automatically segment, detect, and classify CA and AW lesions. The proposed DL model used in the current study is shown in Figure 1. More details about each step are given in the next subsection.

### 2.1. Data Preparation

**Figure 2:**
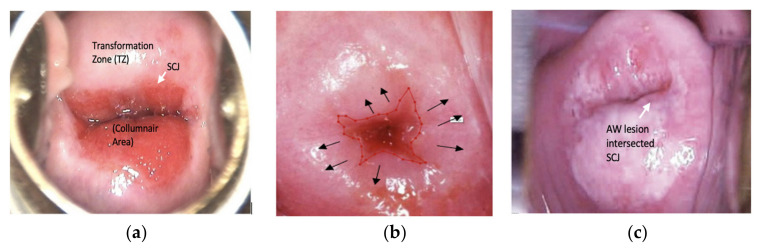
Cervicogram with adequate anatomy landmark. (**a**) Normal condition with the CA, SCJ, and TZ; (**b**) SC junction cells to immature metaplasia; (**c**) abnormal condition with AW lesion intersected with the SCJ.

In the present study, two experienced senior gynecological oncologists defined the important anatomic landmarks to label the cervicogram images. The cervicograms were taken from Mohammad Hoesin Indonesian General Hospital. We performed four steps of preprocessing: (i) collecting digital cervical images; (ii) selecting cervicogram images with adequate pathophysiology and clear AW lesions; (iii) removing unnecessary information with the image cropping process; and (iv) resizing the cervicogram images to produce the same size for all images. The cervicograms were taken using a mobile camera after the application of acetic acid to the cervical region. Due to the utilization of different mobile cameras to capture the cervicograms, the dimensions of the images varied from 150 × 130 pixels to 1024 × 1027 pixels. All cervicograms should have adequate landmarks, such as the CA, subepithelial SCJ region, and AW lesions. Figure 2a shows that red in the endocervix, which is composed of one layer of the columnar cell, appears as the red region, which differs from the TZ, here consisting of multiple layers of squamous cells, which appear as the pink region (Figure 2b). The border between these two different areas is the SCJ. The AW lesion appears as the white area that rises from the SCJ to the TZ (Figure 2c); however, condyloma lesions, closed nabothian glands, and immature metaplasia cells are shaped almost the same as AW lesions but do not coincide with the SCJ.

The dataset was acquired from 484 patients during standard clinical practice in 2020 and 2021 at Mohammad Hoesin General Hospital, Indonesia. A total of 262 patients provided abnormal VIA+ cervicogram images, and 222 patients provided normal VIA− cervicogram images. A whole cervicogram image has a size of about 512 × 512 pixels. The data distribution from the collected cases was split randomly into training, validation, and testing sets for the learning process, as represented in Table 1. Two gynecological oncologist clinicians selected the most widely used landmarks in routine VIA screening. The gynecological oncologists annotated only images complying with the minimal quality requirements, and only a clear cross-sectional scan image was included for further processing. We conducted the learning process without augmentation data because we wanted to maintain the actual clinical conditions.

### 2.2. Image Annotation

**Figure 3:**
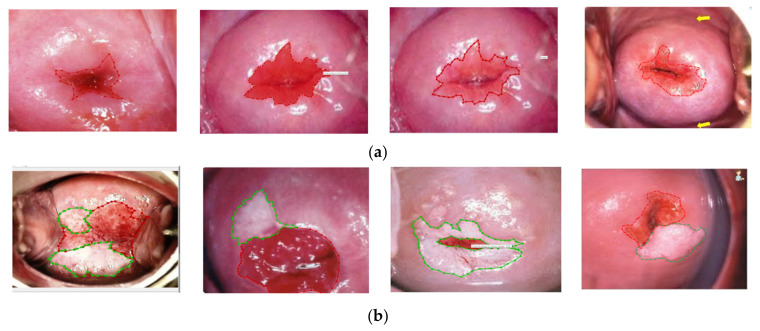
Sample of annotated cervicograms by gynecological oncologist clinicians for standard cervicogram view in (**a**) normal with a red line as the SCJ and red area as the CA; (**b**) abnormal with a green line as the AW lesion and red line as the SCJ.

**Figure 4:**
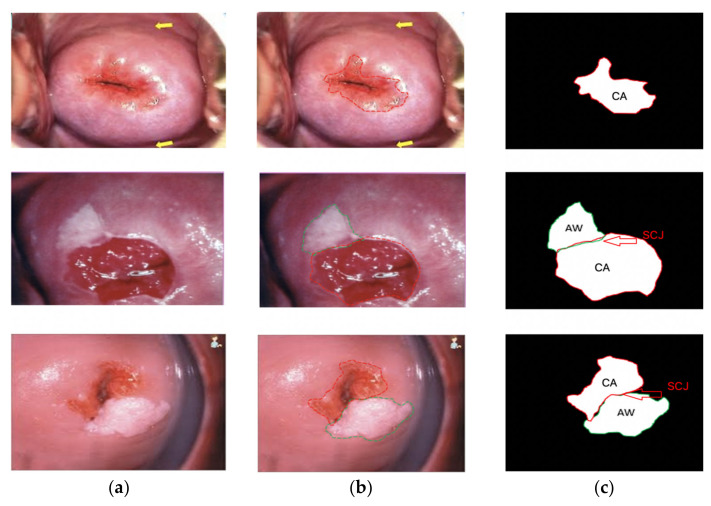
Sample of annotated cervicograms by gynecological oncologists for AW lesion detection (precursor cancer lesion) and normal cervicograms. In the annotation, the region with the red line is the CA, and with the green line is the AW lesion; (**a**) raw data; (**b**) annotation label, and (**c**) squamocolumnar junction (SCJ) forms.

In the segmentation process, the anatomy landmark of the cervix plays an important role, especially in SCJ, CA, TZ, and AW lesions. Two gynecological oncologists who have over 10 years of experience following the above protocol manually annotated such landmarks using an annotation tool (LabelMe) as the ground truth [25,26]. Both normal (without AW lesion) and abnormal (with AW lesion) cervicogram samples had significant variations in image quality, shape, size, and orientation (Figure 3). Annotated images show the CA and AW lesions in the TZ that intersect with the SCJ (Figure 4). Therefore, it is essential to first recognize the SCJ in every image. The whole ground-truth database was saved in JSON file format.

### 2.3. Deep Learning Model

**Figure 5:**
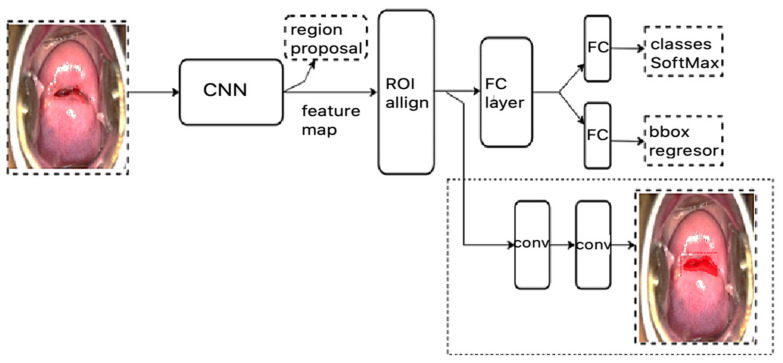
RPNs with the ResNet 50 backbone and FCN architecture for AW lesion detection.

The instance segmentation approach was developed in our previous study and it is based on the mask-RCNN architecture here [25,26]. This architecture has two main parts: the region proposal networks (RPNs) that are used as feature extraction processes from raw data, and fully convolutional networks (FCNs) that function as a multitask learning process for simultaneous classification, detection, and segmentation (Figure 5). The mask-RCNN structure allows for the system to generate the class, location (boundary box), and shape of the object of interest [25,26]. The proposed model utilizes a multitask loss function that combines the loss of classification, localization, and mask segmentation, as illustrated in Equation (1).

(1) $$L_{Total}=L_{\mathrm{cls}}+L_{\mathrm{bbox}}+L_{\mathrm{mask}}$$

The first term is $L_{\mathrm{cls}}$, which measures the error between the ground-truth and predicted class labels. $L_{\mathrm{cls}}$ is the log loss function over the two classes that can be translated from multiclass classification into a binary classification by determining whether the predicted sample is the desired target. $p_{i}^{*}$ is the ground-truth label of anchor box *I*, and $p_{i}$ is the predicted probability of each anchor *i* being an object. The log loss function gives $L_{\mathrm{cls}}$ (Equation (2)).

(2) $$L_{\mathrm{cls}}\;\left(p_{i},p_{i}^{*}\right)=-p_{i}^{*}\log p_{i}-\left(1-{\;p}_{i}^{*}\right)\;\log\left(1-p_{i}\right)$$

The second loss function is $L_{\mathrm{bbox}}$, which measures the difference between $t_{i}^{u}\;$ and $v_{i}$. $t_{i}^{u}\;$ is the predicted result of the bounding box, while $v_{i}$ is the true bounding box. In calculating the loss function $\;\mathrm{of}\;L_{\mathrm{bbox}}$, a smoothing function is added to reduce the sensitivity to outliers. $L_{\mathrm{bbox}}$ and the smoothing function are given by Equations (3) and (4):

(3) $$L_{\mathrm{bbox}}=\underset{i\in x,y,w,h}{\sum }L_{1}^{\mathrm{smooth}}\left(t_{i}^{u}-v_{i}\right)$$

(4) $$L_{1}^{\mathrm{smooth}}=\left\{\begin{array}{cc} 0.5x^{2} & ,\mathrm{if}\;\left|x\right|<1\\ \left|x\right|-0.5 & ,\mathrm{else} \end{array}\right.$$

The third loss is $L_{\mathrm{mask}}$, which calculates the prediction error of the segmentation masks in each instance. The mask branch generates the n × n masking image in every region of interest (RoI) and every class K of the total classes (CA and AW lesions). Hence, the total output was of size K … n^2^. When the model generated the mask image, there was no competition among the classes because the model learnt from a mask from each class. $L_{\mathrm{mask}}$ is the average binary cross-entropy loss only including the k-th mask if the region is associated with ground-truth class k, as defined in Equation (5).

(5) $$L_{\mathrm{mask}}=-\frac{1}{m^{2}}\underset{1\leq i,j\leq m}{\sum }\left[\;y_{ij}log\;{\hat{y}}_{ij}^{k}+\left(\;1-y_{ij}\right)\log\left(1-{\hat{y}}_{ij}^{k}\right)\right]$$

where $y_{ij}$ is the label of a cell (i, j) in the true mask for the region of size m × m, and ${\hat{y}}_{ij}^{k}$ is the predicted value of the same cell in the mask learned for ground-truth class k.

**Figure 6:**
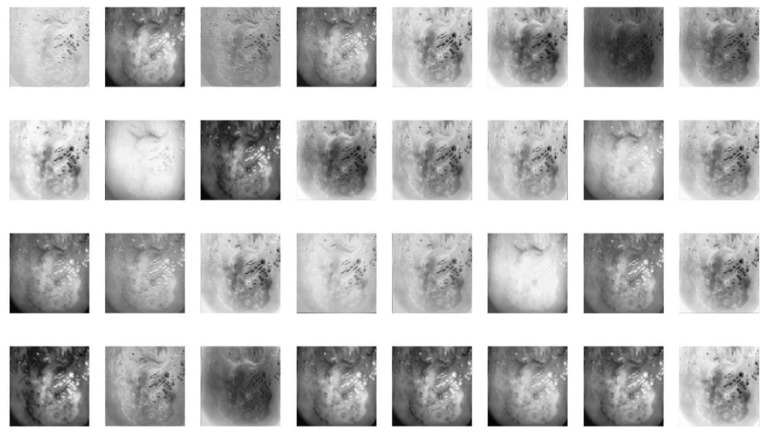
An example of a feature map extracted from the ResNet50 backbone in the RPN.

For the feature extraction mechanism, the ResNet50 architecture was applied as the backbone in the RPNs to produce a feature map, as shown in Figure 6. All feature maps were generated from different residual blocks. These features provide insights into the internal representation of the specific input to be learned by the FCNs. It also helps in understanding why the model might fail to correctly classify some of the images, hence aiding in finetuning the model for better accuracy and precision.

### 2.4. Model Evaluation

To validate and evaluate the performance of the instance segmentation model, the outputs of mask-RCNN were validated by using three metrics: the *IoU*, dice similarity coefficient (DSC) for segmentation, and mean average precision (mAP) for object detection. The *IoU* is a value based on the statistical similarity and diversity of sample sets; its purpose is to evaluate the overlapping (intersecting) area between two bounding boxes, namely, the predicted and ground-truth bounding boxes. The *IoU* is formulated in Equation (6).

(6) $$IoU=\frac{area\;of\;overlap}{area\;of\;union}$$

DSC is a statistical tool that measures the similarity and diversity of sample sets [25,26,27]. In this case, we measured the performance of predictive images with detailed truth labels. The DSC is illustrated in Equation (7).

(7) $$DSC\;\left(X,Y\right)=\frac{2\times area\;of\;Overlap}{Total\;Number\;of\;Pixels}$$

The mAP score is a widely adopted metric for assessing object-detection models. The mAP values of the various groups were computed, and their averages were obtained. Although the model could detect various objects, the classes assigned to these objects were not always certain. However, even if the expected class for an object or instance is correct, the output criterion must still look at how well the model locates it spatially in the picture. Equation (8) depicts the most commonly used mAP.

(8) $$\mathrm{mAP}=\frac{1}{n_{cl}}\;\overset{i=n}{\underset{i=1}{\sum }}AP_{i}$$

where ($n_{cl}$) refers to the total of all the different classes, and $AP_{k}$ is the average precision of class i.

## 3. Results and Discussion

We benchmarked widely used state-of-the-art CNN-based mask-RCNNs with three backbone architectures: ResNet50, ResNet101, and MobileNetV1. The networks’ original architecture of mask-RCNN was maintained in all cervicograms. All networks were first pretrained using the Microsoft Common Objects in Context (COCO) dataset [25,26], and then fully retrained using our training data to produce the probability scores for each class.

### 3.1. Cervicogram Segmentation and Detection Performan

We conducted the segmentation process using a normal cervicogram with the anatomy of CA, SCJ, and TZ, and an abnormal cervicogram with the anatomy of CA, SCJ, and AW lesions. CA and AW lesions were segmented on the basis of lesion contour, and combined with a bounding box with RoI for lesion detection. The whole images were trained and validated using the mask-RCNN architecture, and three performance metrics were used to assess how well such models work, namely, IoU, DSC, and mAP [28].

In the current study, a mask-RCNN with a ResNet 50 backbone was trained with different learning rates (LR) ranging from 0.001 to 0.00001, with 0.1 increments. Every model was trained using the Adam optimizer, 8 batch size, and 50 epochs. The IoU metric assesses how similar the predicted results are compared with the ground-truth label in the range of 0–1. The greater the IoU value is, the more similar the model prediction with the ground truth label; here, the IoU baseline was set at 0.5. Using a learning rate of about 0.0001, the segmentation process achieved satisfying performance for the IoU, 63.61% for the CA, and 72.43% for the AW lesions (Table 2). In comparison, the DSC assesses how similar the predicted results are to the ground truth by measuring the boundary around the feature. The proposed model achieved a DSC of about 72.55% and 88.81% for the CA and AW lesions, respectively (Table 2).

mAP evaluates how well the model detects a desired object. Detection is accurate if the intersection boundary between the ground truth and prediction has a minimal value of 50% with the same label. From the experiment, the proposed model produced a mAP of about 86.90% for the CA and 100% for AW lesions (Table 2). The unique aspect of our study is that it was validated using Herf’s theory, which states that a true AW lesion always intersects with the SCJ. HPV infection spreads from the subepithelial SCJ that expands to TZ, while a false AW lesion, such as a condyloma lesion, closed nabothian gland, and immature metaplasia cells, do not intersect with the SCJ. The experiment showed that our proposed model can detect a white-spot region with abnormal blood vessels in cervicograms by simultaneously classifying and segmenting the CA.

To analyze the proposed model with the ResNet 50 backbone, we evaluated the model and compared it with other architectures, including ResNet 101 and MobileNetV1. The proposed model with ResNet 50 and 0.0001 LR still produced satisfactory performance compared with the other architectures (Table 3) in terms of the IoU, DSC, and mAP. In addition, AW lesion detection reached 100% in mAP, meaning the model could perfectly detect precancerous lesions.

**Figure 7:**
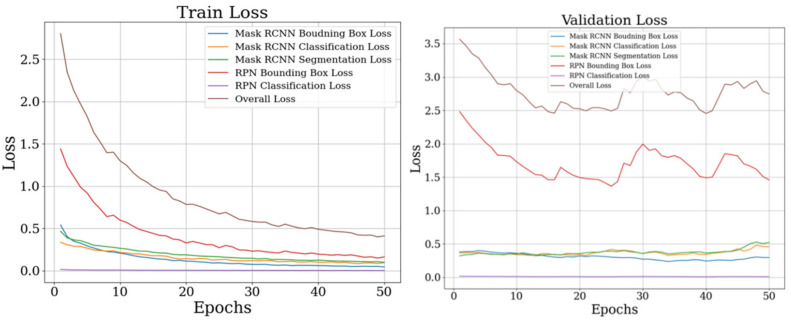
Training and validation loss from the learning process with the ResNet50 backbone.

To find out whether the proposed model could localize objects, recognize each class of objects, and segment lesion contours, training and validation losses were analyzed. There were two losses in the RPNs, three losses in the FCNs, and one average loss (overall loss). Mask-RCNN decouples three tasks in process learning, that is, the bounding-box prediction, the class prediction, and the mask branch, generating mask segmentation for each class without competition among classes. In the present study, the overall loss indicated good performance and converged to one value of about 0.4 in training, and 0.7 in validation (Figure 7).

**Figure 8:**
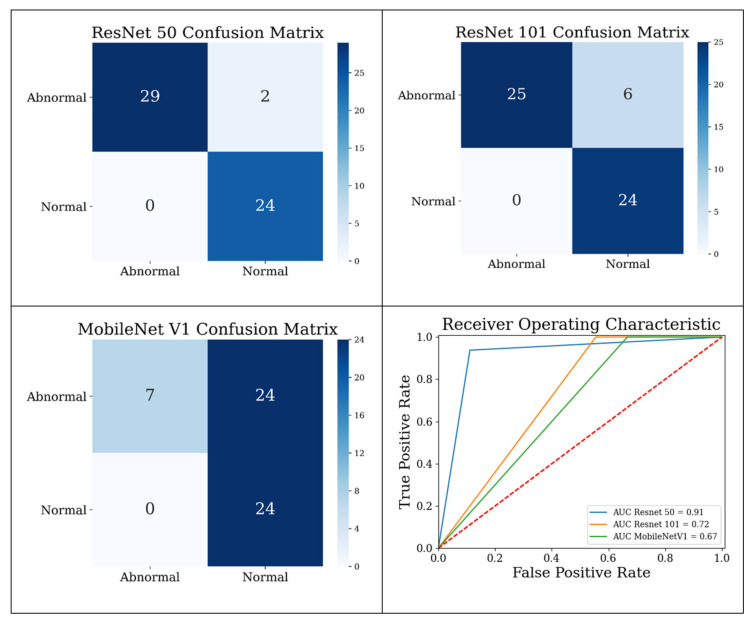
Classification performance with confusion matrix and ROC curve for three backbones.

The classification process was carried out on the basis of two cervicogram images, namely, normal and abnormal anatomical cervical patterns, and the model successfully predicted all normal cervicogram images with 100% sensitivity (Table 4). The classification result based on the ResNet 50 architecture achieved 96.26% accuracy and 92% specificity. Furthermore, the ResNet 50 backbone had the smallest number of false positives and false negatives (Figure 8). A receiver-operating characteristic (ROC) curve was generated to analyze the accuracy of the proposed model. The ROC curve of the ResNet 50 backbone showed the best performance compared with other architectures, with an area under the curve (AUC) value of 0.91 (Figure 8). This means that mask-RCNN could achieve satisfying performance in terms of its segmentation, detection, and classification metrics.

**Figure 9:**
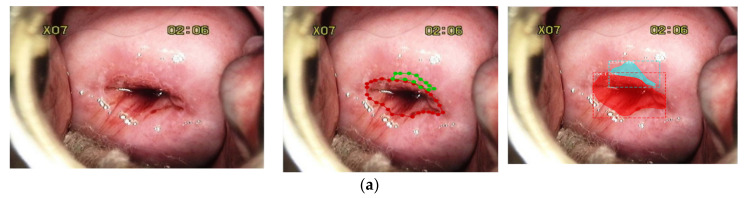

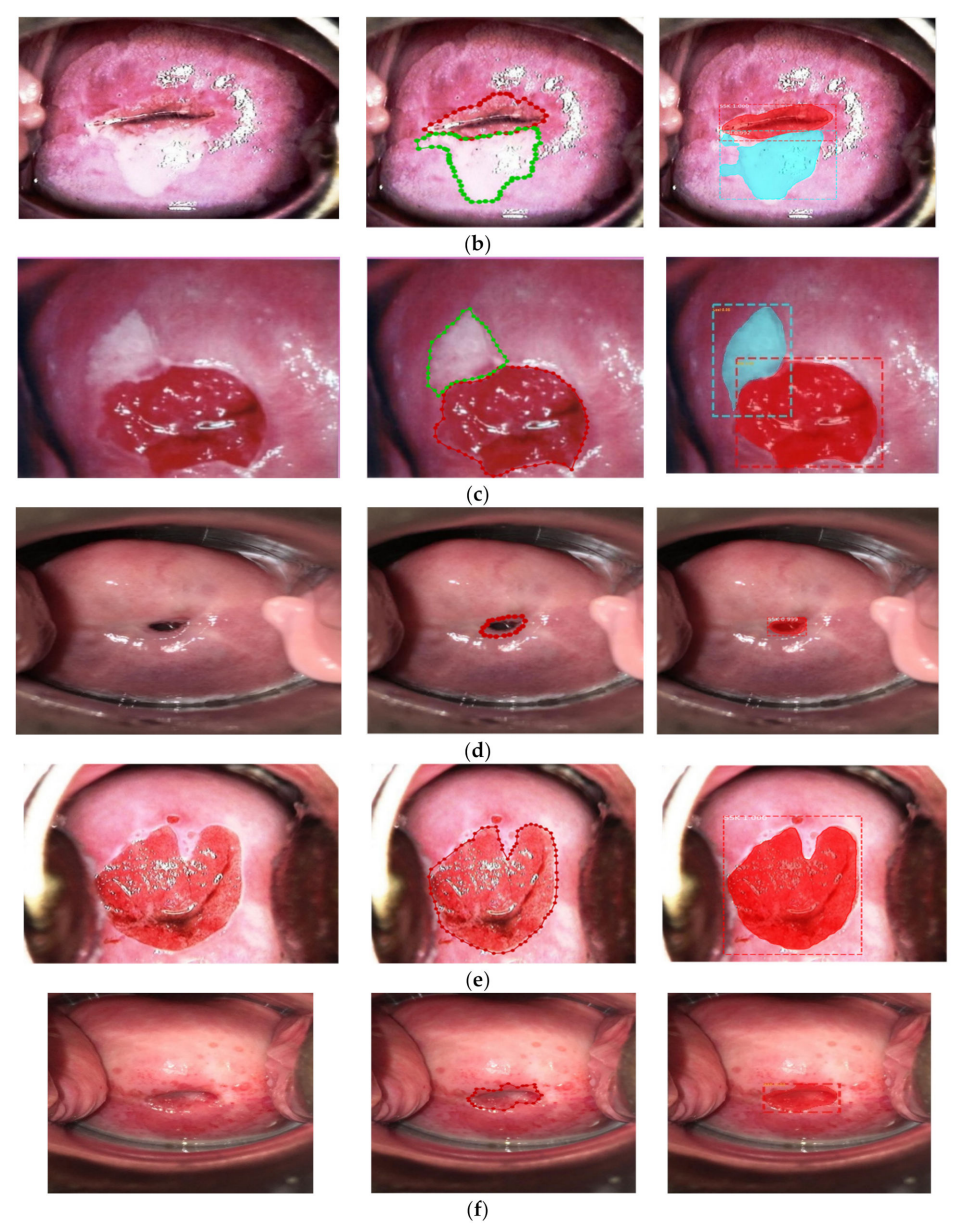
The result of mask-RCNN to identify CA and AW lesions. (left to right) Raw image, annotation image (red line for CA and green line for AW lesions in the ground truth), and prediction of CA and AW lesions (red for CA, and green for AW lesions). (**a**–**c**) Abnormal cervicograms; (**d**–**f**) normal cervicograms.

From the experiment, the cervical area in terms of the columnar epithelial-cell (CA), squamous epithelial-cell (SCJ), and white spot (AW) regions with abnormal blood vessels were segmented and precisely detected. As shown in Figure 9, the proposed model achieved higher detection precision in lesion areas with normal and abnormal conditions. The cervix was fully circular, where the SCJ and TZ can be seen in the four screening quadrants (top right, bottom right, top left, and bottom left). The sample prediction images using the proposed model produced AW lesions showing cervical precancerous lesions or VIA+ in Figure 9a–c, whereas only the CA or VIA− is shown in Figure 9d,e. The lesion area with VIA+ or VIA− stratified squamous cell metaplasia was successfully segmented and detected with a confidence value of 90% and IoU over 50%. Cervical intraepithelial neoplasia (CIN) cells have nuclei that are larger and darker than those of normally mature squamous cells. Greasing with 5% acetic acid causes the cytoplasm to shrink and renders the nucleus more prominent. When light hits the CIN, it is reflected back like a mirror, giving it a white appearance. Normal cells are translucent, allowing for light to enter the veins below, giving the cervix its pink appearance. Columnar cells appear to be red because they are one layer above the vascular tissue.

### 3.2. Benchmarking Our Model with Existing Studies

We benchmarked our proposed DL model with other studies on precancerous prediction. To the best of our knowledge, very limited studies have used DL models based on VIA cervicograms. For a fair comparison, the selected model was benchmarked with the same work area on cervical-cancer precursor-lesion detection with a cervicogram with two-channel inspection (acetic acid and Lugol’s iodine) [27,28] and one-channel inspection (only acetic acid) [29,30]. As shown in Table 5, the summary of our benchmarking is as follows:

- We used a high confidence value of 0.9 and IoU values ranging from 0.5 to 0.7 to ensure that our proposed model provided good results. However, our results still outperformed existing studies using SVM [27], Faster-RCNN [28], and a CNN classifier [23,30]. Two cases used fusion inspection with acetic acid and Lugol’s iodine, but our model only used acetic acid. For the entire IoU value, sensitivity performance was 100%, which means that there were no unclassified results or zero false-negative results.
- HLD-Net developed a detection model with a Faster-RCN architecture with dual-channel fusing detection information across acetic acid and Lugol’s iodine cervicograms [28]; however, this model does not have segmentation capabilities in an infected lesion. AW lesions are hard to detect because their region should intersect with the SCJ in the CA. Using Faster-RCNN achieved sensitivity, specificity, and accuracy of only 40%, 99%, and 69%, respectively. However, our mask-RCNN model was successful in detecting and classifying AW lesions with 98% confidence, 0.5 IoU, 100% sensitivity, 92% specificity, and 96% accuracy to distinguish between normal (without AW lesions) and benign (with AW lesions) tissues.
- Colposcopic images were developed using the SVM algorithm [27] to classify cervicograms on the basis of the corresponding pathology for visual inspection with acetic acid, visual inspection with Lugol’s iodine, and a combination of the two contrasts. However, SVM only achieved 81.3%, 78.6%, and 80% for sensitivity, specificity, and accuracy, respectively.
- The combination of the k-means clustering algorithm and CNN classifier was proposed in [29], and the classification result achieved good performance with 86% accuracy. In [23,30], the CNN based on ResNet 50 architecture was used as the classifier. In [23], it achieved 89% sensitivity and 91% accuracy, while in [30], the accuracy, sensitivity, and specificity were 84.10%, 85.38%, and 82.62%, respectively. However, the CNN classifier only predicts normal and abnormal images, and the position of an infected lesion by HPV cannot be detected. This should be improved by the localization and visualization of AW lesions, so that the results can be explained. Our proposed could perform classification, segmentation, and detection tasks, which means that the results are more reliable for clinical practice.

Nonetheless, the current study has some limitations. First, the 484 pathological still images used in model training, validation, and testing were not enough to ensure that this algorithm was suitable for practical clinical use. A well-designed study using the full cervical-cancer precursor-lesion images of many cases should be conducted to verify the clinical accuracy of the proposed model before the mask-RCNN-based combined model is used in clinical practice. Second, abnormal images present three conditions of CIN, including a precancerous condition in which abnormal cells grow on the surface of the cervix. However, because of image limitations, our models could not be trained on different CIN levels. If sufficient image acquisition was achieved for such lesions, the model could be trained and merged into the existing combined model.

## 4. Conclusions

Deep learning is a data-hungry method, but we showed that a surprisingly small number of cervicogram images could be used to significantly boost diagnosis from what is commonly found in practice. We experimented by selecting the input data according to clinical recommendations for distinguishing anatomy landmarks on the basis of Herf’s theory of the pathogenesis of cervical cancer caused by HPV. This strategy allowed for us to reduce the input data size in our diagnostic model, thereby achieving computational efficiency. Prediction efficiency is key to translating the current study into real-world, resource-poor settings. We developed and validated the mask-RCNN architecture using an instance segmentation approach for cervical-cancer precursor-lesion detection with cervicograms and pathogenesis, which distinguished abnormal cervicograms into VIA+ and VIA−. The proposed model provided effective detection and classified each type of lesion separately with high accuracy. Furthermore, this model demonstrated improved detection ability with a single channel of only acetic acid when compared with another model with a corresponding two-channel pathology for visual inspection with acetic acid and Lugol’s iodine. Our model provides a more reliable lesion detection approach for real-world clinical practice. We look forward to testing and refining these models in larger populations to achieve the more accurate early detection of cervical cancer by medical workers, especially in LMICs.

## Figures and Tables

**Table 1:** Data distribution for the learning process.

Cervicogram	Training	Validation	Testing	Total
Normal	187	15	24	226
Abnormal	206	26	31	263

**Table 2:** Mask-RCNN performance with different learning rates.

Learning Rate	IoU (%)		DSC (%)		mAP (%)	
	CA	AW Lesion	CA	AW Lesion	CA	AW Lesion
0.001	65.65	57.28	73.19	76.94	84.17	99.83
0.0001	63.61	72.43	72.55	88.81	86.90	100
0.00001	37.98	27.81	51.85	53.37	75.42	95.60

**Table 3:** Mask-RCNN performance with three backbone architectures.

Architecture	IoU (%)		DSC (%)		mAP (%)	
	CA Region	AW Lesion	CA Region	AW Lesion	CA Region	AW Lesion
ResNet50	63.61	72.43	72.55	88.81	86.90	100
ResNet101	63.73	72.73	73.22	86.73	83.75	99.85
MobileNetV1	62.38	71.09	66.87	85.06	70.59	72.09

**Table 4:** Mask-RCNN classification performance with three backbone architectures.

Architecture	Performance (%)				
	Accuracy	Sensitivity	Specificity	Precision	F1 Score
ResNet50	96.29	100	92	93.54	96.67
ResNet101	89.10	100	80	80.64	89.29
MobileNetV1	56.36	100	50	22.58	36.84

**Table 5:** Benchmarking results with the existing research on cervical-cancer precursor lesions.

Methods	Learning Process	IoU	Sensitivity	Specificity	Accuracy	Confidence	Inspection
SVM [27]	Classification	-	0.81	0.79	0.80	-	Acetic acid and Lugol’s iodine
Faster-RCNN [28]	Classification and detection	0.2	0.82	0.90	0.86	0.80	Acetic acid and Lugol’s iodine
		0.3	0.63	0.94	0.78	0.80	
		0.4	0.40	0.99	0.69	0.80	
		0.4	0.55	0.67	0.61	0.80	Acetic acid
		0.4	0.49	0.57	0.53	080	Lugol’s iodine
K-means clustering and CNNs classifier [29]	Classification	-	0.84	0.90	0.86	-	Acetic acid
CNN with ResNet 50 [23]	Classification	-	0.89	-	0.91	-	Acetic acid
CNN with ResNet 50 [30]	Classification	-	0.85	82.62	0.84	-	Acetic acid and Lugol’s iodine
Mask-RCNN with ResNet50 (our model)	Segmentation, classification, and detection	0.4	1	0.92	0.96	0.97	Acetic acid
		0.5	1	0.92	0.96	0.97	
		0.6	1	0.77	0.87	0.98	
		0.7	1	0.50	0.60	0.98	

## Data Availability

The data is available by request.
